# The Major Chromosome Condensation Factors Smc, HBsu, and Gyrase in Bacillus subtilis Operate via Strikingly Different Patterns of Motion

**DOI:** 10.1128/mSphere.00817-20

**Published:** 2020-09-09

**Authors:** Sonja Schibany, Rebecca Hinrichs, Rogelio Hernández-Tamayo, Peter L. Graumann

**Affiliations:** a SYNMIKRO, Loewe-Zentrum für Synthetische Mikrobiologie, Philipps-Universität Marburg, Marburg, Germany; b Fachbereich Chemie, Philipps-Universität Marburg, Marburg, Germany; University of Iowa

**Keywords:** *Bacillus subtilis*, DNA gyrase, HBsu, SMC, chromosome condensation, histone-like protein

## Abstract

Although DNA-compacting proteins have been extensively characterized *in vitro*, knowledge of their DNA binding dynamics *in vivo* is greatly lacking. We have employed single-molecule tracking to characterize the motion of the three major chromosome compaction factors in Bacillus subtilis, Smc (structural maintenance of chromosomes) proteins, topoisomerase DNA gyrase, and histone-like protein HBsu. We show that these three proteins display strikingly different patterns of interaction with DNA; while Smc displays two mobility fractions, one static and one moving through the chromosome in a constrained manner, gyrase operates as a single slow-mobility fraction, suggesting that all gyrase molecules are catalytically actively engaged in DNA binding. Conversely, bacterial histone-like protein HBsu moves through the nucleoid as a larger, slow-mobility fraction and a smaller, high-mobility fraction, with both fractions having relatively short dwell times. Turnover within the SMC complex that makes up the static fraction is shown to be important for its function in chromosome compaction. Our report reveals that chromosome compaction in bacteria can occur via fast, transient interactions *in vivo*, avoiding clashes with RNA and DNA polymerases.

**IMPORTANCE** All types of cells need to compact their chromosomes containing their genomic information several-thousand-fold in order to fit into the cell. In eukaryotes, histones achieve a major degree of compaction and bind very tightly to DNA such that they need to be actively removed to allow access of polymerases to the DNA. Bacteria have evolved a basic, highly dynamic system of DNA compaction, accommodating rapid adaptability to changes in environmental conditions. We show that the Bacillus subtilis histone-like protein HBsu exchanges on DNA on a millisecond scale and moves through the entire nucleoid containing the genome as a slow-mobility fraction and a dynamic fraction, both having short dwell times. Thus, HBsu achieves compaction via short and transient DNA binding, thereby allowing rapid access of DNA replication or transcription factors to DNA. Topoisomerase gyrase and B. subtilis Smc show different interactions with DNA *in vivo*, displaying continuous loading or unloading from DNA, or using two fractions, one moving through the genome and one statically bound on a time scale of minutes, respectively, revealing three different modes of DNA compaction *in vivo*.

## Abstract (precis)

All types of cells need to compact their chromosomes containing their genomic information several-thousand-fold in order to fit into the cell. In eukaryotes, histones achieve a major degree of compaction and bind very tightly to DNA such that they need to be actively removed to allow access of polymerases to the DNA. Bacteria have evolved a basic, highly dynamic system of DNA compaction, accommodating rapid adaptability to changes in environmental conditions. We show that the Bacillus subtilis histone-like protein HBsu exchanges on DNA on a millisecond scale and moves through the entire nucleoid containing the genome as a slow-mobility fraction and a dynamic fraction, both having short dwell times. Thus, HBsu achieves compaction via short and transient DNA binding, thereby allowing rapid access of DNA replication or transcription factors to DNA. Topoisomerase gyrase and B. subtilis Smc show different interactions with DNA *in vivo*, displaying continuous loading or unloading from DNA, or using two fractions, one moving through the genome and one statically bound on a time scale of minutes, respectively, revealing three different modes of DNA compaction *in vivo*.

## INTRODUCTION

In living cells, the genetic material is not distributed randomly but is compacted by a combination of macromolecular crowding, DNA-architectural protein activity, and supercoiling. How this compaction is achieved has been a matter of debate for decades (1, 2).

However, an understanding of DNA compaction and its relation to dynamic processes such as transcription, replication, recombination, and DNA repair is crucial, since many diseases such as cancer and Cornelia de Lange syndrome are associated with pathological alterations of the three-dimensional (3D) architecture of the genetic material.

Smc (structural maintenance of chromosomes) is a DNA-architectural protein which is conserved in all branches of life and therefore even more ancient than nucleosomes (3). Smc proteins have recently attracted much scientific attention due to the hypothesis that they are able to compact DNA via the process of ATP-driven loop extrusion (4–6). They are very large proteins with an unusual structure: they form dimers that can be closed into a ring structure by the activity of their essential kleisin subunit (ScpA in bacteria), which in turn interacts with a second essential protein, usually a heat repeat-type protein (ScpB in bacteria). In Bacillus subtilis, Smc molecules are distributed in two fractions in living cells: an immobile fraction, likely tightly bound to DNA, and a fraction that moves through the chromosome in a constrained manner (7, 8). However, it is currently unclear which of these fractions mediates chromosome compaction or whether both do and how these fractions change relative to the cell cycle. Additionally, although it was shown previously that ScpA and ScpB downregulate the ATPase activity of Smc *in vitro* (9), the role of the subunits in modulating the activity of Smc *in vivo* remains unclear.

Another DNA-architectural protein is the prokaryotic DNA gyrase (called “gyrase” here). Gyrase belongs to the type 2 topoisomerases, which break double-stranded DNA in an ATP-dependent manner. It passes another double-stranded DNA molecule through this break and thereby changes the linking number of DNA by minus 2 (10). It is a tetramer consisting of two subunits of GyrA and two subunits of GyrB. The whole complex has a size of 326 kDa. Prokaryotic gyrase is an important drug target, since inhibition of its action allows selective killing of bacteria. Antibiotics such as the aminocoumarins (including novobiocin) and quinolones (including nalidixic acid and ciprofloxacin) are all based on gyrase inhibition (11). It is therefore also crucial from a medical point of view to obtain a better understanding of the mode of interaction of gyrase with DNA *in vivo*.

A third player in the maintenance of DNA compaction in B. subtilis is the histone-like protein HBsu. HBsu belongs to the HU family of nucleoid-associated proteins (NAPs). It has been suggested that these proteins are functional homologs of histones, despite the absence of sequence or structural homology (12). Like histones, HBsu engages nonspecifically with DNA and its compaction ability is regulated by acetylation (13). Therefore, it has been hypothesized that a histone-like code exists also in prokaryotes (13). HBsu’s role in DNA compaction has been extensively characterized *in vitro* (14, 15). In one *in vivo* study, HBsu was described as uniformly distributed on the nucleoids (16). Other than this, nothing is known about its DNA binding behavior *in vivo*. In contrast, it was previously shown that histones display a remarkably long residence time on DNA (in the range of minutes) in live eukaryotic cells (17). This residence time changes during developmental processes (18), allowing dynamic regulation of transcription.

In this study, we sought to explore the interactions of these three important DNA-architectural proteins with DNA *in vivo* by using single-molecule tracking (SMT) (7). Our results reveal that the interaction of Smc with DNA is not cell cycle regulated as in eukaryotic cells. This finding suggests that the action of Smc is important throughout the cell cycle and not only for resolving origin regions at the beginning of the cell cycle as previously suggested (19). Furthermore, we found that a fusion of Smc with ScpA was still recruited to “foci” which contain several Smc, ScpA, and ScpB molecules and are statically positioned at several places on the nucleoids. The Smc-ScpA fusion also formed a dynamic fraction, revealing that the preformed Smc-ScpA complex can still interact with DNA *in vivo*. We also found that the residence time of Smc is regulated by its subunit ScpA. In contrast to Smc, the subunit GyrA of gyrase formed only a single, slow-moving fraction on the nucleoid and we could not detect a significant freely diffusing fraction, suggesting that most GyrA molecules are engaged in maintaining supercoiling homeostasis throughout the cell. For the histone-like protein HBsu, we detected a slow-mobility fraction and a dynamic fraction and both fractions displayed a remarkably short residence time at our employed exposure time. These results show that proteins are able to compact DNA via transient interactions and not only by stably engaging with DNA and that bacterial histone-like proteins function in a way not presenting an obstacle for polymerases.

## RESULTS

### Smc and its localization relative to origin regions.

**FIG 1:**
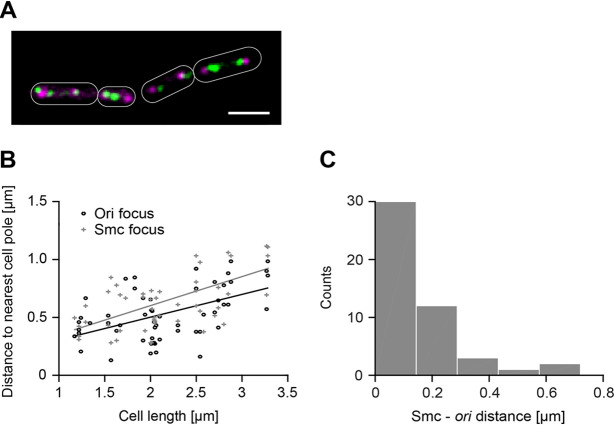
(A) Overlay of cells expressing Smc-mVenus (green) and *ori*-CFP (purple). The scale bar corresponds to 2 μm. (B) Analysis of the distance from *ori*-CFP foci and Smc-mVenus foci to the nearest cell pole (dependent on cell length). (C) Histogram of distance of *ori*-CFP focus to next Smc-mVenus focus (mean = 0.19 μm ± 0.18 μm [standard deviation {SD}]).

Previously, Smc had been described to be clustered around *oriC* regions, forming foci that colocalize with *oriC* and with ParB foci. However, using single-molecule tracking, we recently found that Smc forms immobile clusters at many sites on the chromosome (8). To clarify this issue, we constructed a strain in which a functional Smc-mVenus (Smc-mVenus fluorescent protein) fusion can be localized relative to origin regions. These regions were marked using a *lacO* array to which LacI-CFP (LacI-cyan fluorescent protein) molecules bind. Visual inspection of the CFP and the mVenus signal by using a long exposure time (500 ms) revealed regularly spaced origins in each cell half as reported previously under our growth conditions (Fig. 1A). In contrast, mVenus signals were often diffuse in the cell and often more Smc-mVenus clusters than origins were observed in a cell half. These findings confirm our observation from SMT that Smc stops at sites also outside the origin region. We quantified the distance from the *ori*-CFP foci and Smc-mVenus foci to the nearest cell pole as a function of cell length. Interestingly, we found a clear correlation for both kinds of foci (for *ori-*CFP, rho = 0.505, *P < *0.001, *n* = 56; for Smc-mVenus, rho = 0.570, *P < *0.001, *n* = 51 [Pearson correlation coefficient]), indicating a tendency of *ori*-CFP foci and Smc-mVenus foci to migrate away from the cell poles as cells became elongated and therefore during the progression of the cell cycle (Fig. 1B). An analysis of the distance from the centroids of *ori*-CFP foci to the next Smc foci revealed a mean distance of 0.19 ± 0.18 μm (Fig. 1C), showing further that Smc foci apparently do not always colocalize with origin foci (we found colocalization in 43.4% of foci) and revealing that Smc is also statically bound to DNA away from origin regions. This is consistent with chromatin immunoprecipitation with microarray technology (ChIP-chip) data revealing Smc enrichment at several sites away from *oriC* (20) and with the results of our previous SMT experiments (8).

### Smc diffusion is altered after arrest of transcription or a change in supercoiling.

**FIG 2:**
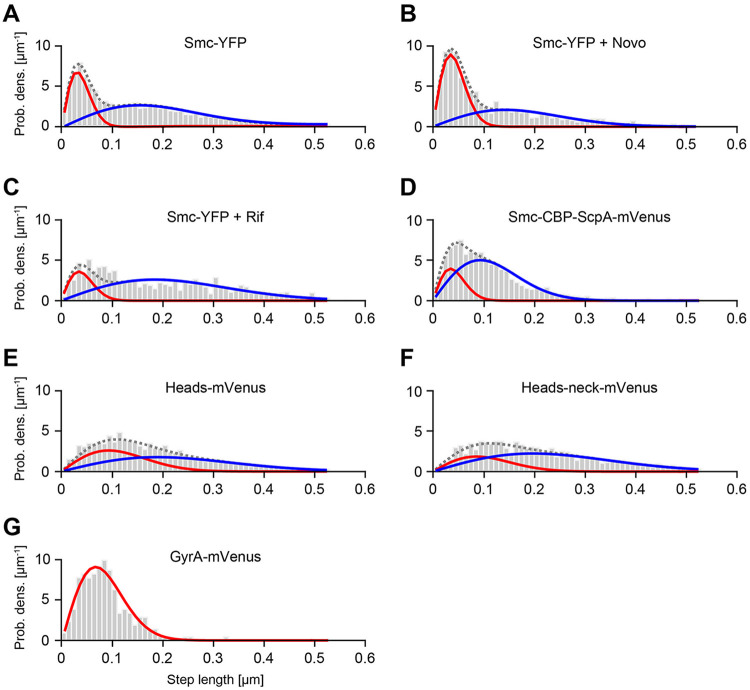
Distribution of step lengths (jump distances) for Smc-YFP, Smc-YFP plus novobiocin, Smc-YFP plus rifampin, Smc-CBP-ScpA-mVenus, Heads-mVenus, Heads-neck-mVenus, and GyrA-mVenus. Plots show the probability density (Prob. dens.) function of steps observed with a certain length. The distributions of the step lengths of GyrA-mVenus (panel G) can be explained by a single Rayleigh distribution, corresponding to a single diffusion constant, while the distribution of Smc can be explained only by two Rayleigh distributions with distinct diffusion constants (red and blue curves). Gray dotted curves indicate the fit to data taking together the two distinct Rayleigh distributions. Molecules were tracked using 30 ms exposure time (A to D), tracked using 7.4 ms exposure time (E to F), and tracked using 10 ms exposure time (G). Data for Fig. 2A are also contained in Schibany et al., 2018 (8).

**FIG 3:**
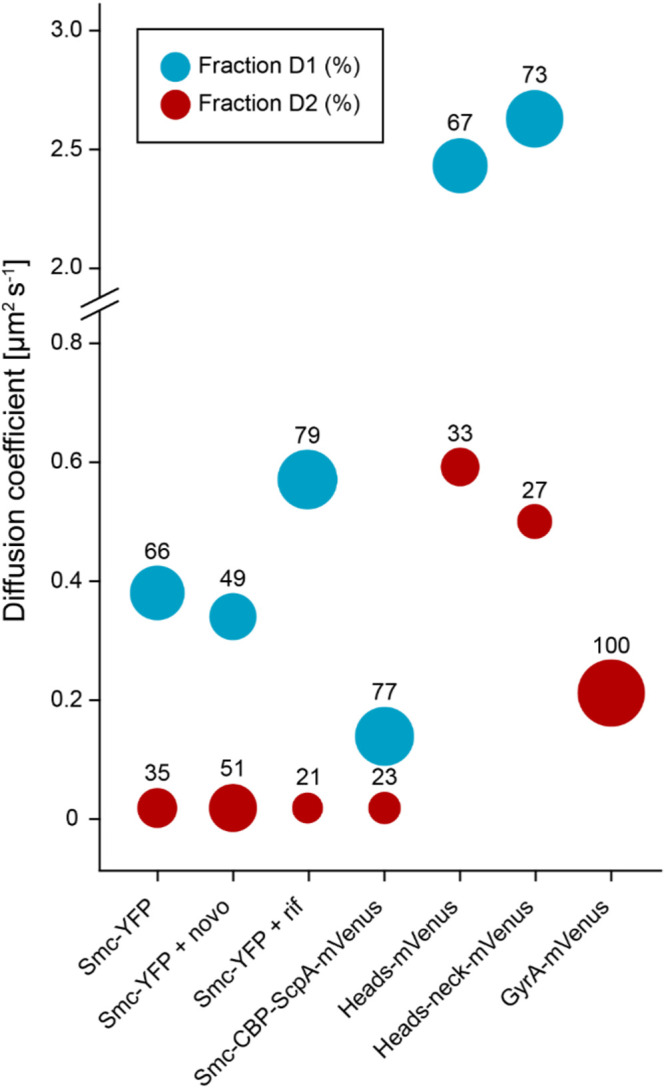
Bubble plot of the determined diffusion coefficients. Numbers indicate sizes of populations in percentages. D1 corresponds to the mobile fraction (corresponding to the blue curves in Fig. 2), D2 to the slow mobile/static (red) fractions shown in Fig. 2. novo, novobiocin; rif, rifampin.

Single-molecule dynamics were captured using “slim-field” illumination (7, 8), and tracks were automatically detected based on the Crocker and Grier algorithm (21) and were analyzed using jump distance (JD) analysis, in which the probability is plotted with which steps of a certain length were taken by molecules. Shown in Fig. 2 are results of JD analyses, in which the probability of small steps (slow-moving molecules) can be seen to be close to zero and that of mobile molecules not close to zero. The distribution of steps observed for Smc cannot be explained by a single population, i.e., with a single Rayleigh distribution (Fig. 2A). Rather, Smc-YFP (Smc-yellow fluorescent protein) shows two distinct mobility fractions as analyzed by SMT: one static/immobile fraction accounting for about 35% of the steps (accounted for by the red Rayleigh distribution fit), and one dynamic population that moves throughout the chromosome (blue distribution fit) (7, 8) (Fig. 2A; see also Fig. 3). Taken together, the two fits can explain the JD distribution of Smc very well (Fig. 2A).

Because the SMC complex functions together with topoisomerases in chromosome compaction (22, 23), we wished to investigate the dynamics of Smc that occur when the DNA topology is altered. Smc-YFP cells were treated for 1 h with the antibiotic novobiocin (10 μg/ml), which inhibits DNA gyrase, the major enzyme generating overall negative supercoiling in the cell. As expected, 90% ± 2% of the cells (three independent experiments, *n* = 69 cells) showed highly decondensed and extended nucleoids and the cells were elongated, likely due to the induction of the SOS response. Background fluorescence was higher in treated cells than in nontreated cells, and from our visual inspection it was clear that the diffusion of Smc was heavily altered. We observed an increase in the proportion of the static fraction of Smc to 51% compared to 35% without novobiocin treatment (Fig. 2B and 3; see also Table 1). This experiment clearly showed that movement of Smc was influenced by supercoiling, either directly or indirectly.

**TABLE 1:** Summary of determined diffusion coefficients^*a*^

Protein fusion	MM (kDa)	Diffusion coefficient (μm^2^ s^−1^)	Fraction (%)
Smc-YFP	159	D1: 0.38	66
		D2: 0.02	35
Smc-YFP + rifampin	159	D1: 0.57	79
		D2: 0.02	21
Smc-YFP + novobiocin	159	D1: 0.34	49
		D2: 0.02	51
Smc-CBP-ScpA-mVenus	198	D1: 0.14	77
		D2: 0.02	23
Heads-mVenus	63	D1: 2.43	67
		D2: 0.59	33
Heads-neck-mVenus	76	D1: 2.63	73
		D2: 0.50	27
GyrA-mVenus	119	D2: 0.21	100
HBsu-mVenus	37	D1: 0.72	38
		D2: 0.09	63

a D1, mobile fraction; D2, slow-mobility/static fraction; MM, molecular mass.

It was elegantly shown previously that the cohesin complex is relocated by RNA polymerase (RNAP) to sites of convergent transcription in the yeast genome (24). We wished to analyze whether the bacterial Smc movement might also be driven at least in part by RNA polymerase translocation. We therefore inhibited RNA polymerase activity by adding 200 μg/ml of the transcription inhibitor rifampin for 30 min. Under these conditions, we observed that the B. subtilis chromosome was decondensed, filling the entire cytoplasm of cells as observed before by others (three independent experiments, *n* = 89) (6). The diffusion of Smc was quantitatively altered (Fig. 2C). In contrast to the results seen with novobiocin treatment, we observed a reduction of the proportion of the native static faction of Smc to 21% (Fig. 3; see also Table 1) compared to 35% without rifampin treatment and a significant increase in the diffusion of mobile Smc, which most likely was caused by the nucleoid decompaction. These findings are in agreement with the idea that movement of Smc depends on constrained diffusion through the chromosome.

### Smc diffusion is independent of the cell cycle.

It was shown previously that Smc is important for the initial separation of origin regions during rapid growth (19) but not for rapid separation of origin regions under slow-growth conditions (25). We therefore wondered if the movements of Smc could differ in a manner dependent on the stage of the cell cycle. Under our growth conditions, cells commenced the cell cycle mostly with two origins that were then segregated toward opposite cell poles before initiation of replication occurred and, finally, cell division (i.e., the replication rounds overlapped slightly). Because cell size roughly corresponds to the stage of the cell cycle, we grouped cells from exponentially growing cultures into three categories: small (<2.25-μm-diameter) cells, medium-sized (2.25-μm to 3.2-μm) cells, and large (>3.2-μm) cells. Interestingly, we did not observe a significant change in the diffusion coefficients or in the percentages of the fractions of static and dynamic molecules (see Fig. S1 at https://doi.org/10.6084/m9.figshare.12818375 [all supplemental files can be found at that URL]). These experiments showed that there was no detectable change in Smc dynamics during the cell cycle. Therefore, the activity of Smc with respect to the static and dynamic fractions appears to be required continuously throughout a cell’s lifetime and not only during the initial segregation of origins to maintain nucleoid organization. There was no significant recruitment of the dynamic pool to stationary molecules during the cell cycle, indicating that both fractions are important to maintain chromosome compaction. This finding is in contrast to the behavior of cohesin in eukaryotes, where cohesin is loaded in a cell cycle-dependent manner and cohesion is established in a manner dependent on replication (26).

### An Smc-ScpA fusion is still recruited to condensation centers but not isolated head domains.

**FIG 4:**
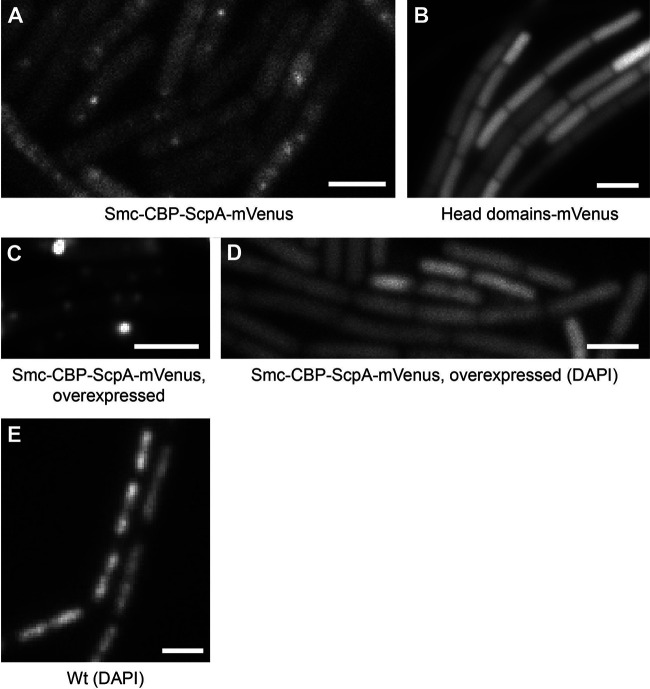
Epifluorescence microscopy of strains expressing (A) an Smc-CBP-ScpA-mVenus fusion induced at low levels (comparable to wild-type Smc levels), (B) a Heads-mVenus fusion, or (C) an Smc-CBP-ScpA-mVenus fusion (overexpressed; 0.5% xylose). (D) DAPI chromosome stain in treated cells overexpressing Smc-CBP-ScpA-mVenus. (E) DAPI chromosome stain in wild-type (Wt) cells. The scale bars correspond to 2 μm.

To shed light on the requirements for the formation of static condensation centers, we generated an Smc-CBP (Smc–calmodulin-binding protein)-ScpA-mVenus fusion (the CBP is used as a spacer between Smc and ScpA; the Smc-CBP-ScpA fusion was kindly provided by Stephan Gruber, Lausanne, Switzerland). Previously, it was shown that this fusion is still functional when expressed as an additional copy on the Bacillus subtilis chromosome and is able to form functional dimers with Smc (27). Low levels of expression of Smc-CBP-ScpA-mVenus did not significantly alter the chromosome structure according to results of comparisons of the chromosome morphology corresponding to DAPI (4′,6-diamidino-2-phenylindole)-stained cells to that of wild-type cells (data not shown). Epifluorescence investigations (exposure time, 500 ms) revealed that 93% ± 6% of the cells still had fluorescent foci, albeit the spacing was not as regular as that seen with Smc-YFP (Fig. 4; compare with Fig. 1). Single-molecule tracking revealed that this fusion still formed a static fraction as well as a dynamic fraction that moved significantly slower than that of Smc-YFP (Fig. 2D and 3; see also Table 1). These experiments revealed that Smc-ScpA was still recruited to condensation centers *in vivo* and showed reduced mobility. This reduced mobility was probably due to the increased hydrodynamic radius of the fusion compared to Smc. In contrast to the results seen with low-level induction, a high level of induction (0.5% xylose) led to the formation of brighter clusters and decondensed chromosomes (Fig. 4). This is in strong contrast to the overexpression of wild-type Smc, which results in chromosome hypercompaction (28). These experiments indicated that, like, e.g., the transition state mutation in Smc in B. subtilis and Caulobacter crescentus (29, 30), the Smc-ScpA fusion represents a dominant-negative result. Thus, when the Smc-CBP-ScpA fusion was expressed in excess of Smc, it interfered with chromosome compaction *in vivo*.

We next asked if isolated head domains can still be recruited to condensation centers, since we observed that a Headless-mVenus fusion was no longer recruited to these structures (8). Therefore, we connected the N- and C-terminal domains of *smc* with a linker, fused this head-domain construct to *mVenus*, and expressed the fusion ectopically. Single-molecule tracking revealed two dynamic fractions: one corresponding to a velocity of 0.59 ± 0.41 μm^2^ s^−1^ (33%) and the other to a velocity of 2.43 ± 0.72 μm^2^ s^−1^ (67%) (Fig. 2E; see also Fig. 3). Importantly, no static fraction was observed, a finding which was also confirmed using epifluorescence microscopy (Fig. 4).

It was shown previously that the neck region of Smc binds also to ScpA (27). We therefore wondered if inclusion of the neck region adjacent to the head domains could significantly reduce the diffusion of this fusion. However, single-molecule tracking again showed two diffusing fractions, one with 0.50 ± 0.19 μm^2^ s^−1^ (27%) and the other with 2.63 ± 0.50 μm^2^ s^−1^ (73%) (Fig. 3; see also Table 1). Thus, our results show that there was no significant difference between these two Smc-head-domains fusions with respect to diffusion. Inclusion of the neck region did not lead to a reduction of the diffusion constant *in vivo*, although the neck regions interacted with ScpA *in vitro*.

### Induction of an Smc-ScpA fusion reduces turnover of condensation centers.

**FIG 5:**
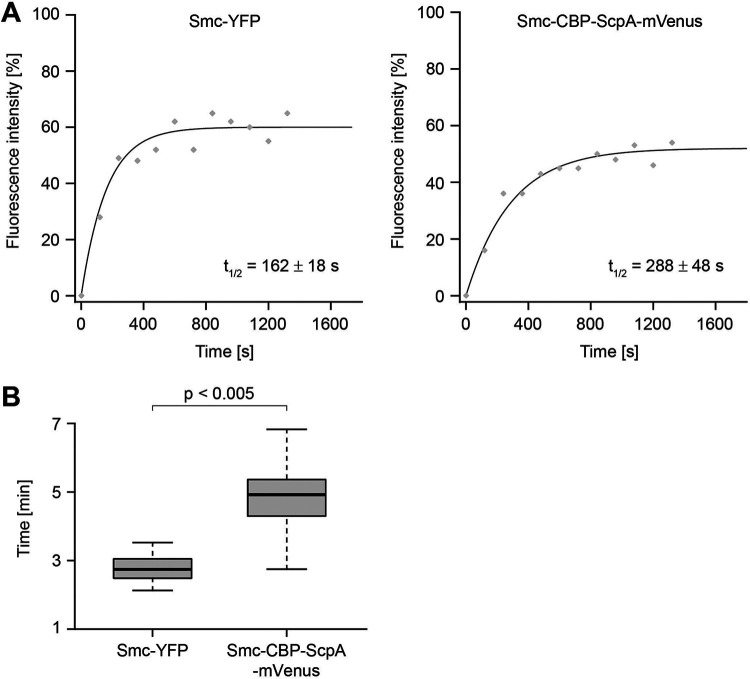
FRAP analysis of exponentially growing cells. (A) Fluorescence intensity (%) plotted over time (s) for Smc-YFP (left) and Smc-CBP-ScpA (right). The line represents the fit used to calculate the recovery half-time. The calculated recovery half-time for Smc-YFP is 162 ± 18 s and that for Smc-CBP-ScpA-mVenus is 288 ± 48 s. (B) Box plot showing the distribution of calculated recovery half-times for Smc-YFP and Smc-CBP-ScpA-mVenus. Smc-YFP and Smc-CBP-ScpA-mVenus display significantly different residence times (Student's *t* test, *P < *0.005).

We wished to investigate further if the direct fusion of Smc and ScpA has an effect on the dwell time within condensation centers. Therefore, we employed fluorescence recovery after photobleaching (FRAP) experiments, which yield quantitative information about the residence time of fluorescent protein fusions (31). We previously showed that Smc-GFP has a half-time turnover of 2.7 ± 0.56 (standard error [SE]) min whereas that of ScpA is 4.7 ± 0.61 (SE) min (30). Here, we found a significantly prolonged residence time of Smc-ScpA (half-time recovery of 4.8 min ± 48 s) in comparison to Smc (2.7 min ± 18 s) under conditions in which Smc-CBP-ScpA was expressed at a level similar to that seen with Smc-YFP (Fig. 5). These data indicate that opening of the Smc/ScpA connection partially regulated Smc turnover within the centers. This result provides a possible explanation for our observation that overexpression of Smc-ScpA affects proper chromosome compaction. Thus, the turnover of Smc is important for correct chromosome compaction and segregation.

### Functionally active gyrase shows slow mobility throughout the chromosome in B. subtilis.

We further wanted to compare the diffusion characteristics of Smc to those of another protein that is involved in DNA architecture. We chose DNA gyrase, which is a key player in maintaining supercoiling homeostasis in B. subtilis (32) and other prokaryotes (33).

As with Smc, we expressed GyrA-mVenus from its original gene locus that was under control of the native promoter and followed single molecules using a 10-ms exposure time. In contrast to Smc, the step lengths could not be sorted into two fractions, but a one-component fit already explained our data sufficiently well (Fig. 2G). We determined a diffusion coefficient of 0.21 ± 0.06 μm^2^ s^−1^. This diffusion coefficient was not determined according to localization precision; thus, gyrase was not entirely immobile in the cells, like the static Smc fraction, but diffused very slowly and engaged stably with DNA *in vivo*. We interpret the results as indicating that this fraction represents molecules undergoing catalysis *in vivo*. Thus, the whole pool of gyrase appears to be engaged in maintaining supercoiling homeostasis *in vivo*, and no considerable free pool of GyrA could be detected. The residence time of GyrA-mVenus was determined by photobleaching performed as described for Smc; therefore, we could not determine its dwell time using single-molecule tracking (30).

**FIG 6:**
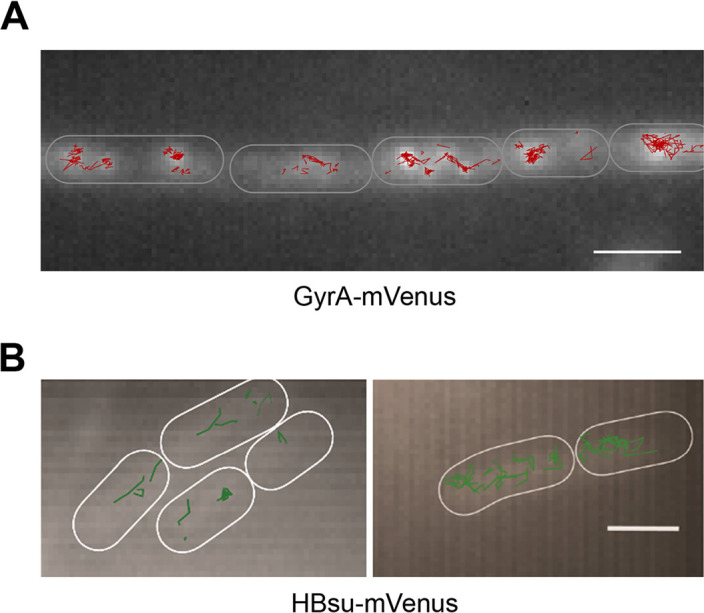
2D track analysis. (A) Tracks of GyrA-mVenus that were classified as representing slow diffusion overlaid on the average projection of the whole stream. These tracks could be found throughout the cells (outlines indicated by white ovals) and not only concentrated at the sites of replisomes. (B) Tracks of HBsu-mVenus within cells (the outline is indicated by the white ovals). The scale bars correspond to 2 μm.

We further looked at the distribution of GyrA molecules in the cells and found that they were not concentrated in a single focus but were instead distributed throughout the cells (Fig. 6A), in accordance with a role in maintaining supercoiling homeostasis throughout the cell and not only at a single point. However, we could also observe an apparent clustering resembling the previously described centers adjacent to replisomes (32, 33) (Fig. 6A).

### A DNA-architectural protein compacts DNA via transient interactions *in vivo*.

To investigate the interaction with DNA of a third DNA-architectural protein, we chose the histone-like protein HBsu. The role of this protein and of other nucleoid-associated proteins (NAPs) in DNA compaction has been investigated extensively both *in vitro* (34, 35) and *in vivo* (13), but its mode of interaction with DNA has not been investigated *in vivo*.

**FIG 7:**
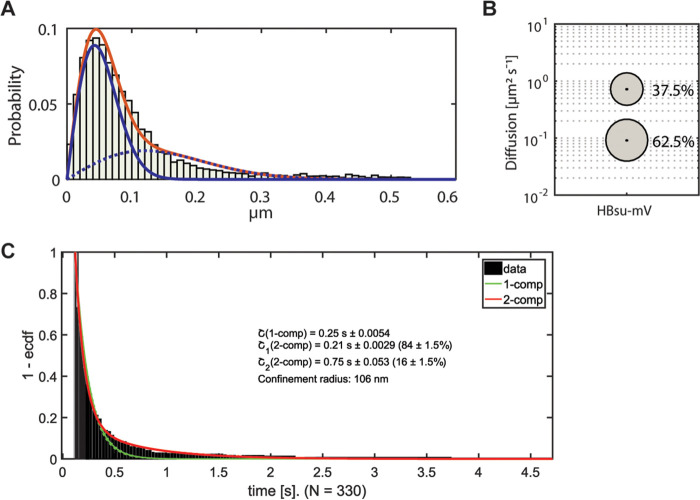
Jump distance analysis and dwell times for HBsu-mVenus. (A) Jump distance analysis showing a two-population fit of step lengths of HBsu-mVenus. Dashed blue line, Rayleigh fit to the fast-mobility population; solid blue line, Rayleigh fit to the slow-mobility fraction. The red solid line shows combined fits. Tracking was done using 10 ms exposure time. (B) Bubble plot showing fraction sizes of slow-mobility and fast-mobility populations. (C) Dwell time analyses using a confinement radius of 106 nm (corresponding to the pixel size of the camera). The green line shows a one-component fit and red line a two-component fit. Tracking was done using 30 ms exposure time. ecdf, empirical cumulative distribution function.

We expressed an HBsu-GFP fusion from its native promoter in minimal medium. We confirmed that the fluorescence tag did not interfere with the function of HBsu, because the growth rate was not significantly different from that of the wild-type strain (see Fig. S2A). As a second test, we scored the compaction state of the nucleoids and calculated a score similar to that seen with the wild-type cells (see Fig. S2B). Since HBsu-GFP is a rather small protein (monomer, 37 kDa; dimer, 74 kDa), we expected that the fusion would be trackable only with a rather short exposure time. Indeed, 10 ms of exposure time resulted in circular point spread functions. Because we wanted to employ similar fluorophores for all proteins and avoid effects of a long bleaching time or a low level of brightness of the fluorophore, we constructed an HBsu-mVenus fusion that was integrated into the *amyE* site on the chromosome. We performed induction with low levels of xylose (0.01%), which resulted in only one or two simultaneously expressed copies, as evidenced by the low number of signals seen in the results of the experiments. As shown in Fig. 7A, two Rayleigh distributions were required to explain the observed jump distance distribution, with an *R*^2^ value of 0.9989, which is very close to a value of 1. Jump distance analyses showed a diffusion constant of 0.091 ± 0.001 μm^2^/s for the slow-mobility fraction, which comprised 62.5% ± 0.003% of the step lengths, and a diffusion constant of 0.719 ± 0.008 μm^2^/s for the fast-moving fraction, which comprised 37.5% ± 0.003%. While the slow-mobility fraction of HBsu moved with a coefficient similar to that of the slow-mobility fraction of Smc (8), the dynamic fraction still moved more than five times more slowly than free mVenus (27 kDa) in B. subtilis (8). Because HBsu is only 10 kDa in size, even a homodimer of HBsu-mVenus would be expected to have a much higher diffusion coefficient, suggesting constrained movement of HBsu due to transient interactions with DNA. Thus, HBsu displays a stationary/slow-mobility fraction like the other DNA-architectural proteins Smc (7), MukB (36), and CTCF (37) and the DNA repair protein RecN (38), as well as a dynamic fraction, both of which move throughout the nucleoid (Fig. 6B). This finding is also in line with results from conventional epifluorescence microscopy where HBsu showed fluorescence throughout the nucleoids (39, 40).

To further quantify the interaction of HBsu-mVenus with DNA, we determined its *in vivo* dwell times. Of note, actual residence times *in vivo* are underestimated by our approach, because our analyses employ bleaching of fluorescent protein fusions. In order to reduce interference from photobleaching, we tracked HBsu-mVenus with 30 ms of integration time. We calculated the survival function by determining the time during which molecules rested within a circle of radius 106 nm. Our analysis revealed that HBsu displayed two fractions, one with a dwell time of ∼210 ms (84% of molecules, τ_1_) (Fig. 7C) and a second, much smaller fraction of 16% with 750 ms dwell time (τ_2_). Of note, with an average track length of 7.9 steps (i.e., of 240 ms) from our acquisitions, the estimate of τ_1_ does not suffer from a high level of interference from photobleaching, whereas that for τ_2_ does. However, with an average track length of about 8 steps, the number of long tracks was sufficient to allow us to be reasonably confident with the estimated time of 750 ms. In any event, a large majority of HBsu molecules showed short dwell times and thus transient interactions with likely DNA *in vivo*. Thus, whereas Smc is stationary on DNA and gyrase moves very slowly *in vivo*, this is not the case for the DNA compaction protein HBsu, which performs its DNA-architectural role by two types of transient interactions. It has been suggested that the NAPs of the HU family are functional homologs of histones despite the absence of structural homology (12). However, HU protein has been shown to bind to DNA transiently by *in vitro* experiments (44), and our work shows that the modes of interaction with DNA of HBsu and of histones are markedly different *in vivo*. Possibly, the residence time of HBsu is reduced by the process of facilitated dissociation (41–43) *in vivo* and could therefore be entirely different from that determined *in vitro*. Thus, HBsu molecules perform their DNA-architectural or catalytic function with short residence times and not on a time scale of several minutes, as was shown extensively for histones and other DNA-architectural proteins (17).

## DISCUSSION

Chromosomes have to be compacted about 1,000-fold in bacterial cells, and much more in eukaryotic cells, and yet the DNA has to remain accessible for RNA polymerase, transcription factors, DNA repair proteins, and the replication forks. We show that three major compaction factors in the bacterial model system B. subtilis achieve their task in markedly different modes of operation. While the very-low-abundance Smc protein (about 30 copies per origin region) moves through the entire chromosome in a constrained motion and forms several static, tightly DNA-bound centers (between 4 and 8 per genome) together with the SMC complex partners ScpA and ScpB, DNA gyrase moves through the chromosome in a slow manner, possibly constantly binding/catalyzing strand passage and releasing DNA. Although gyrase can accumulate at replication forks (32, 33), we show that in general, gyrase is spatially stochastically localized on the entire chromosome. The finding that a freely diffusive fraction of gyrase is missing (or any second population of gyrase that diffuses faster than the one observed) suggests that very soon after gyrase has dissociated from its last place of action, it finds another place without longer periods of searching. In contrast, 65% of Smc proteins spends many minutes in a diffusive state and 35% in a tightly DNA-bound state for an average of 2.5 minutes, as shown by FRAP experiments. A third pattern of DNA association with the genome is found for the nucleoid-associated protein HBsu, a small basic protein. HBsu diffuses as two populations, a large slow-mobility fraction of about 63% and a fast-mobility fraction of 37%. Even the fast-mobility fraction moves with a diffusion coefficient much lower than would be expected for a small soluble protein (38), indicating that all of the detectable HBsu molecules moved through the nucleoid in a constrained manner. A large fraction of 84% of the molecules had a relatively short average dwell time of 210 ms; only 16% showed a residence time of close to 1 s. These findings suggest a high degree of fast unbinding and rebinding kinetics. Therefore, based on its high on and off rates, this highly abundant protein poses no strong obstacle for RNA polymerase or DNA polymerases, and it can be concluded that it achieves chromosome compaction via direct binding on a time scale of milliseconds. Clearly, HBsu operates in a way very different from that of histones, which need to be modified and mechanically moved for free access to DNA. In agreement with our findings, rapid and transient binding to DNA has been shown for the Escherichia coli histone-like protein HU *in vitro* (44). Possibly, the residence time of HBsu is further reduced by the process of facilitated dissociation *in vivo* (41–43), conceivably accounting for the differences *in vitro* and *in vivo*. Thus, bacteria appear to have evolved a simple mechanism for compaction using high on and off rates for a nucleoid-associated protein.

Being of low abundance, Smc is unlikely to represent an obstacle for DNA and RNA polymerases. Smc requires head domains for its function and for binding to ScpA and ScpB, while hinge and coiled coil domains (or a dimer) are sufficient for constrained motion through the nucleoid (8). Because the ATPase domain that might power Smc movement resides in the head domains, we wondered how Smc moves through the nucleoid. We tested if inhibition of RNA polymerase would slow or abolish Smc movement, but the contrary was true; Smc became faster in its movement and less statically positioned. In contrast, inhibition of DNA gyrase led to slowing of Smc motion, and more Smc molecules became static at the expense of mobile molecules. These experiments support the idea that dynamic Smc molecules diffuse through the chromosome by Brownian motion and yet do so in a constrained manner, by nonspecific interaction of coiled coil domains with the DNA. This interaction is clearly influenced by the structure of the DNA, as RNAP or gyrase inhibition considerably affected Smc dynamics. Extending these analyses, we asked if isolated Smc head domains would show any interaction with the chromosome, for which we found no evidence. Circumstantial evidence suggests that some head domain molecules may move along with ScpAB, while most molecules freely diffuse, based on our detection of two rapidly diffusing populations. ScpAB molecules diffuse through the entire cell as a complex, with a diffusion constant of about 1.2 μm^2^ s^−1^, and upon binding to isolated head domains may slow to the observed 0.5 μm^2^ s^−1^ rate. In any event, head domains were not recruited into static SMC centers, but interestingly, a fusion of Smc and ScpA was recruited to these structures, of which there appear to be between 2 to 4 per origin region. Mild induction of the Smc-ScpA fusion did not generate any phenotype, but strong induction led to chromosome decondensation, while overproduction of wild-type Smc results in chromosome hypercondensation (28). The fusion of Smc to ScpA showed a considerably increased average life time within the static SMC centers, as found using FRAP analyses, suggesting that Smc’s inability to let go of ScpA increases its time spent in the static mode. These experiments suggest that an average *k*_off_ rate for complex formation is important for the function of Smc. Possibly, a prolonged dwelling of Smc on DNA disturbs dynamic processes of replication, transcription, and DNA repair, which all rely on movement of chromosome segments and DNA accessibility (45). Perturbing these dynamic processes may then indirectly lead to chromosome compaction and segregation defects. Note that we found a half-life time of about 2.5 min for wild-type Smc, suggesting that after this time, Smc is released from its tightly DNA bound state to diffuse through the chromosome. Smc is therefore not expected to dwell for longer times on DNA and thus would not be able to travel along DNA from *oriC* toward the terminus for longer periods of the cell cycle as suggested by a recent model on zipping up chromosome arms by extended SMC movement (6). Furthermore, it is possible that it is not only the stationary fraction of Smc that mediates DNA compaction but also the dynamic one, which has so far been disregarded with regard to its functional importance. Transient interactions observed for eukaryotic cohesin and condensin may also confer compaction activity.

Whereas several traits like those represented by the basic residues are shared between eukaryotic histones and HBsu, our study results show that the interactions with DNA are very different. Whereas histones engage stably with DNA on a timescale of minutes, HBsu shows transient binding and yet confers compaction throughout the genome. At present, we cannot explain the basis of the two observed fractions of HBsu; it will be interesting to determine if HBsu has different exchange rates at different sites on the nucleoids.

Interestingly, HBsu is acetylated *in vivo* and this acetylation influences DNA compaction (13). Possibly, the fraction of acetylated HBsu and therefore its residence time are altered in differentiated cells, as in competent and sporulating cells. A reduced residence time of HBsu in competent cells would facilitate access of the incoming DNA for recombination by decreasing DNA compaction. On the other hand, it is possible that HBsu interacts more stably with DNA during sporulation to secure tight DNA compaction and protection. Thus, it should be investigated in future studies whether there is a link between compaction state and residence time of this NAP.

Our results are compatible with a ChIP-chip study using HBsu-myc (39). In that study, it was found that the binding profile of HBsu-myc was much more uniform than that of the protein Rok, which showed visible clustering in epifluorescence microscopy. None of the chromosomal regions analyzed had >2.5-fold enrichment for HBsu-myc, consistent with the function of HBsu as a nonspecific nucleoid binding protein and with a very short binding time on all parts of the chromosome as was found in our study. An alternative interpretation is that the residence time of HBsu is too low to obtain a statistically significant ChiP-chip signal, leading to the rather uniform distribution of binding sites.

Bacterial chromosomes have been shown to be comprised of relatively well-defined microdomains, or chromosomal interaction domains (CIDs), which were detected using Hi-C (46–48). Nucleoid-associated proteins FIS and H-NS have been suggested to be involved in the generation of domains in E. coli (49, 50). Due to population averaging over many cells in Hi-C, nothing can be deduced about CID dynamics at timescales of milliseconds to minutes. If we assume that HBsu also plays a role in CID-boundary formation, such domain boundaries could be very dynamic entities, based on our findings. It was recently found by direct imaging that domain boundaries in the E. coli genome are influenced by the NAPs HU, H-NS, and Fis (51); therefore, rapid changes of the 3D architecture of the nucleoid that depend on environmental conditions or differentiation states may allow rapid changes in gene expression to occur, enabled by fast NAP dynamics.

In summary, our data show that a single NAP, HBsu, compacts an entire genome through transient interactions occurring in two modes, a slow-mobility and a fast-moving manner, avoiding steric clashes with polymerases, as well as the need for extensive modifications for mobilization, while DNA gyrase and Smc proteins operate via distinct dynamics, medium-temporal binding for gyrase and very-short-term as well as long-term binding by Smc, here involving only very few molecules. Thus, bacteria have evolved a much simpler and yet efficient set of proteins that mediate compaction than eukaryotes, using either very low copy numbers or high turnover rates for DNA binding.

## MATERIALS AND METHODS

### Cell growth and preparation.

Bacillus subtilis strains were grown in S7_50_ defined medium. For induction of the xylose promoter, glucose was exchanged for 0.5% fructose, and the proportion of xylose was increased to 0.05% for a low level of induction and to 0.5% for overexpression. For induction of the *spac* promoter, the culture media were supplemented with 1 mM isopropyl β-d-1-thiogalactopyranoside (IPTG). Antibiotics (chloramphenicol [5 μg*/*ml], erythromycin [0.5 μg*/*ml], lincomycin [12.5 μg*/*ml], or spectinomycin [50 μg*/*ml]) were used when necessary. Overnight cultures were grown at 21°C in S7_50_ medium, resulting in cells growing exponentially the next day.

### Strain construction.

To obtain strains SS130, SS126, and SS128, PCR-amplified parts of the *smc* gene were cloned in 1193NLMV vector (lab stock) via isothermal assembly (52) using oligonucleotides listed in Table S1 and transformed into competent B. subtilis PY79. Expression of *smc-cbp-scpA-mVenus*, *heads-mVenus*, and *heads-neck-mVenus* was confirmed by in-gel fluorescence (see Fig. S3 and S4 [all supplemental files can be found at https://doi.org/10.6084/m9.figshare.12818375]). Strain GyrA-mVenus was created by cloning a 500-bp fragment containing the 3′ portion of the *gyrA* gene into 1164NLMV vector (lab stock) (53), again via isothermal assembly (52). The assembled partly sequenced plasmid was transformed into competent B. subtilis PY79 (see Table S2). The *hBsu-mVenus* fusion was constructed from a gene fragment containing the entire *hns* gene (encoding HBsu) plus a ribosome binding site, an SGSLGD linker, and the *mVenus* sequence (BioCat GmbH Heidelberg), cloned into pSG1193 (54) using ApaI and SpeI sites. B. subtilis BG214 was transformed with the resulting construct.

### Single-molecule tracking and data acquisition.

Cells were dropped onto a coverslip, and an agarose pad (1% agarose mixed in S7_50_ medium) was put on top to supply the bacteria with nutrients and prevent them from drying up. Cells were imaged with a high-numerical-aperture (high-NA) lens objective (CFI Plan Apo Lambda DM 100× oil; Nikon) (NA = 1.49) on a Nikon Eclipse Ti microscope equipped with a back-illuminated electron multiplying charged-coupled-device (EMCCD) camera (Hamamatsu ImageEM X2). An EMCCD gain of 200 was used. Illumination was achieved by focusing the excitation laser light onto the objective’s back focal plane. A laser power density of ∼160 W cm^−2^ was used for single-molecule tracking using 30 ms exposure time (33 Hz) and of ∼310 W cm^−2^ for tracking at 100 Hz and at 135 Hz. For fast-moving molecules, a shorter exposure time was used, since we observed cloud-like structures or elliptical point spread functions using 30 ms exposure time, which is expected for this time scale in a bacterial cell for relatively fast-diffusing molecules (55, 56). The fluorescence in the cells was bleached until only one to two diffusing molecules in the cell were detectable to avoid spuriously connected positions of single molecules. In each movie, 1,500 frames were acquired, resulting in no detectable growth defects after this illumination period (data not shown). Imaging was performed at 21°C. At least 100 tracks were scored for each analysis.

### Single-molecule tracking and track analysis.

Images were recorded using VisiView (Visitron Systems) and were subsequently analyzed by using ImageJ software version 1.50f (W. Rasband, National Institutes of Health, Bethesda, MD, USA). For particle tracking, published analysis software was used (57). The tracks were manually checked. Single molecules were identified and trajectories of single molecules were constructed using MATLABtrack software (57). Trajectory analysis was done with this software and also with custom software written in MATLAB. Importantly, for ectopically expressed proteins, the initial brightness of a cell was determined before image processing and, to avoid effects from overexpression of the protein of interest, only cells with numbers of molecules similar to the number of Smc-YFP molecules were analyzed. The diffusion coefficient for the static fraction of Smc-YFP, Smc-YFP plus rifampin, Smc-YFP plus novobiocin, and Smc-CBP-ScpA-mVenus was constrained to 0.02 μm^2^ s^−1^. For HBsu-mVenus, SMTracker 1.5 was used (58).

### Measurement of diffusion coefficient dependent on the cell cycle.

Cell lengths were determined from bright-field images. To select cells of certain lengths, proper regions of interest (ROIs) were created in MATLABTrack (57).

### FRAP analysis.

Fluorescence recovery after photobleaching (FRAP) measurements were done as described previously by Schenk et al. (59).

### Measurement of dwell time.

For the determination of the dwell time, SMTracker 1.5 (https://sourceforge.net/projects/singlemoleculetracker/) was used (58, 60).
